# Delayed intubation associated with in-hospital mortality in patients with COVID-19 respiratory failure who fail heated and humified high flow nasal canula

**DOI:** 10.1186/s12871-023-02198-7

**Published:** 2023-07-12

**Authors:** Christian Bime, Gordon E. Carr, Jie PU, Sherri Kou, Ying Wang, Michael Simons

**Affiliations:** 1 University of Arizona College of Medicine, Tucson, AZ USA; 2 Banner Health, Phoenix, AZ USA

## Abstract

**Background:**

Advanced respiratory support modalities such as non-invasive positive pressure ventilation (NiPPV) and heated and humidified high flow nasal canula (HFNC) served as useful alternatives to invasive mechanical ventilatory support for acute respiratory failure (ARF) during the peak of the SARS-CoV-2/COVID-19 pandemic. Unlike NiPPV, HFNC is a newer modality and its role in the treatment of patients with severe ARF is not yet clearly defined. Furthermore, the characteristics of responders versus non-responders to HFNC have not been determined. Although recent evidence indicates that many patients with ARF treated with HFNC survive without needing intubation, those who fail and are subsequently intubated have worse outcomes. Given that prolonged use of HFNC in patients with ARF might exacerbate patient self-inflicted lung injury, we hypothesized that among those patients with ARF due to COVID-19 pneumonia, prolonged HFNC beyond 24 h before intubation would be associated with increased in-hospital mortality.

**Methods:**

This was a retrospective, multicenter, observational cohort study of 2720 patients treated for ARF secondary to SARS-CoV-2/COVID-19 pneumonia and initially managed with HFNC within the Banner Health system during the period from March 1^st^, 2020, to July 31^st^, 2021. In the subgroup of patients for went from HFNC to IMV, we assessed the effect of the duration of HFNC prior to intubation on mortality.

**Results:**

1392 (51%) were successfully treated with HFNC alone and 1328 (49%) failed HFNC and were intubated (HFNC to IMV). When adjusted for the covariates, HFNC duration less than 24 h prior to intubation was significantly associated with reduced mortality.

**Conclusions:**

Among patients with ARF due to COVID-19 pneumonia who fail HFNC, delay of intubation beyond 24 h is associated with increased mortality

**Supplementary Information:**

The online version contains supplementary material available at 10.1186/s12871-023-02198-7.

## Introduction

During the peak of the SARS-CoV-2/COVID-19 pandemic, there was a dramatic increase in the demand for advanced respiratory support modalities such as non-invasive positive pressure ventilation (NiPPV), heated and humidified high flow nasal canula (HFNC), as alternatives to invasive mechanical ventilation (IMV) [1]. In the early phase of the pandemic, concerns about potential aerosolization of SARS-CoV-2 viral particles and healthcare personnel infection limited the use of NiPPV and HFNC for patients presenting with acute respiratory failure (ARF) due to COVID-19 pneumonia [2–6]. However, during subsequent waves of the pandemic, there was a better appreciation of the transmission risk associated with SARS-CoV-2 hence a gradual and sustained increased in the use of these non-invasive modalities for ARF [7–10]. Increased capacity strain on healthcare systems caused by the influx of critically ill patients together with intensive care unit (ICU) resource limitations led to further increases in use of NiPPV and HFNC for patients with ARF as alternatives to invasive mechanical ventilation [8, 10].

Although the standard of care for patients with acute respiratory failure (ARF) has traditionally been early IMV with lung protective strategies [11], it has long been recognized that some patients might be more appropriate for non-invasive modalities such as NiPPV or HFNC [12–14]. For example, in patients with ARF due to acute exacerbations of chronic obstructive pulmonary disease (COPD) or congestive heart failure (HF) with pulmonary edema, early non-invasive positive pressure ventilation (NiPPV) has been shown to reduce rates of intubation and improve survival [13, 14]. Unlike NiPPV, HFNC is a newer modality for treatment of ARF and its role in the treatment of patients is not yet clearly defined [12]. Furthermore, the characteristics of responders versus non-responders have not been determined [12, 15–18]. Current clinical practice guidelines strongly recommend a trial of HFNC in patients with ARF with hypoxemia [19], however the subset of patients presenting with ARF who are ideal for trial of HFNC has not been defined. Nor has the optimal timing of HFNC before considering intubation and invasive mechanical ventilation (IMC) been defined. We previously showed, using data prior to the COVID-19 pandemic, that among patients with ARF, those who failed NiPPV or HFNC and were subsequently intubated had a significantly increased mortality compared to IMV alone [12]. Studies have shown that the ratio of oxygen saturation as measured by pulse oximetry/fraction of inspired oxygen to respiratory rate (ROX index) of less than 5.99 (ARF due to COVID-19) [20] and less than 4.88 (non-COVID-19 ARF) [21] at 12 h post initiation is a good predictor of HFNC failure [22]. Nevertheless, the optimal duration of an HFNC trial prior to endotracheal intubation and IMV is not clear. In patients with severe ARF with poor lung compliance, prolonged use of HFNC might exacerbate patient self-inflicted lung injury (SILI) [23]. SILI might lead to physiologically difficult intubations and possibly negatively affect other organs such as the brain, the heart, and the kidneys. We hypothesized that among those patients with ARF due to COVID-19 pneumonia who failed a trial of HFNC and were subsequently intubated, a delay of intubation beyond 24 h would be associated with increased in-hospital mortality. Older age is a major risk factor for COVID-19 pneumonia severity and mortality [24]. Therefore, we wanted to test for effect modification by age on the response to HFNC among patients with ARF due to COVID-19.

## Methods

### Study population

This was a retrospective, multicenter, observational cohort study of subjects treated for ARF secondary to SARS-CoV-2/COVID-19 pneumonia and initially managed with HFNC within the Banner Health system during the period from March 1^st^, 2020, to July 31^st^, 2021. Patients were included for this analysis if they were ≥ 18 years old, had a laboratory-confirmed diagnosis of COVID-19 by qualitive polymerase-chain-reaction (PCR) assay, and had acute hypoxemic respiratory failure treated with HFNC for ≥ 2 h prior to any use of IMV. Patients were excluded if NiPPV was the first manner of advanced respiratory support and those for whom endotracheal intubation was not within their goals of care. All data were collected from the electronic medical record. The Institutional Review Board of Banner Health (IRB#483–20-0076) approved this study.

Banner Health System’s protocol for the management of acute respiratory failure due to COVID-19allowed the use of alternatives to IMV (HFNC and NiPPV), however, their use was not encouraged outside of those patients who were “do not intubate” status prior to the initiation of advanced respiratory support. Both HFNC and NiPPV were listed as “second line” therapies (compared to IMV), which when implemented, required close monitoring, with a recommendation for rapid intubation if signs of failure were noted. For HFNC, the ROX index was recommended as one component of follow-up evaluation and failure determination, though this was not mandatory. Our protocol did not have mandatory criteria for initiation of HFNC and determination of failure was determined by the bedside intensive care team. Although the initial protocols required negative pressure isolation with an appropriately fitted N95 mask or alternative for staff caring for SARS-CoV-2/COVID-19 pneumonia on HFNC and NiPPV, this requirement was later rescinded for HFNC when updated information indicated that it was not necessary.

The Banner Health System critical care clinical consensus group met at least monthly during this period to review the available literature and update guidance for the care of these patients.

### Statistical analysis

Patients were separated into two groups based on the clinical trajectory and respiratory support modality used. One group was successfully treated with HFNC alone (HFNC success) and the other group was treated first with HFNC then transitioned to IMV (HFNC failure). Mortality was ascertained based on discharge disposition (alive or dead). We used Person’s chi-square test of independence and odds ratio with 95% confidence intervals to test and compare differences in mortality between the HFNC success group and the HFNC failure group. We next focused on the HFNC failure group to assess the effect of the duration of HFNC prior to intubation on mortality. Logistic regression was used to build the multivariable model with in-hospital mortality as the outcome. Ten explanatory variables are selected based on the testing result in univariate analysis. Factors with *p*-value < 0.05 in the univariate analysis were selected as explanatory variable in the multivariable models. Since data was not collected in a prospective manner, the multivariable model developed could only be used to test for an association between the independent variables and the outcome. We included the following covariates: age, sex, comorbidities (chronic obstructive pulmonary disease [COPD], heart failure [HF], Diabetes mellitus [DM], chronic kidney disease [CKD], hypertension[HTN]), baseline laboratory values (C Reactive Protein [CRP] (> 100 mg/L or <  = 100 mg/L), Troponin I (> 28% or <  = 28%), NT–proBNP (> 88 pg/mL or <  = 88 pg/mL), Lymphocyte Count (> = 1.0 µL or < 1.0 µL) and Creatinine (< = 1.5 mg/dL or > 1.5 mg/dL), and duration of HFNC prior to intubation in the univariate and multivariate analysis. We included CRP and NT–proBNP as covariates in the final model given well known positive associations with COVID-19 severity and mortality [25, 26]. The baseline laboratory values were obtained prior to initiation HFNC.

The first comparison was overall in-hospital mortality between HFNC group and HFNC to IMV group. We next focused on HFNC to IMV group to assess the impact of duration of HFNC prior to intubation on in-hospital mortality. We conducted a univariate analysis for each of the variables with in-hospital mortality. Next a multivariable logistic regression model was developed with in-hospital mortality as the outcome and the following covariates: sex, age, DM, CKD, hypertension, Troponin > 28, Creatinine > 1.5, HFNC duration prior to intubation as covariates.

To assess for effect modification, we repeated the univariate and multivariate analysis after stratification by age group: age18 to 49 years old and age 50 and older.

## Results

**Fig. 1:**
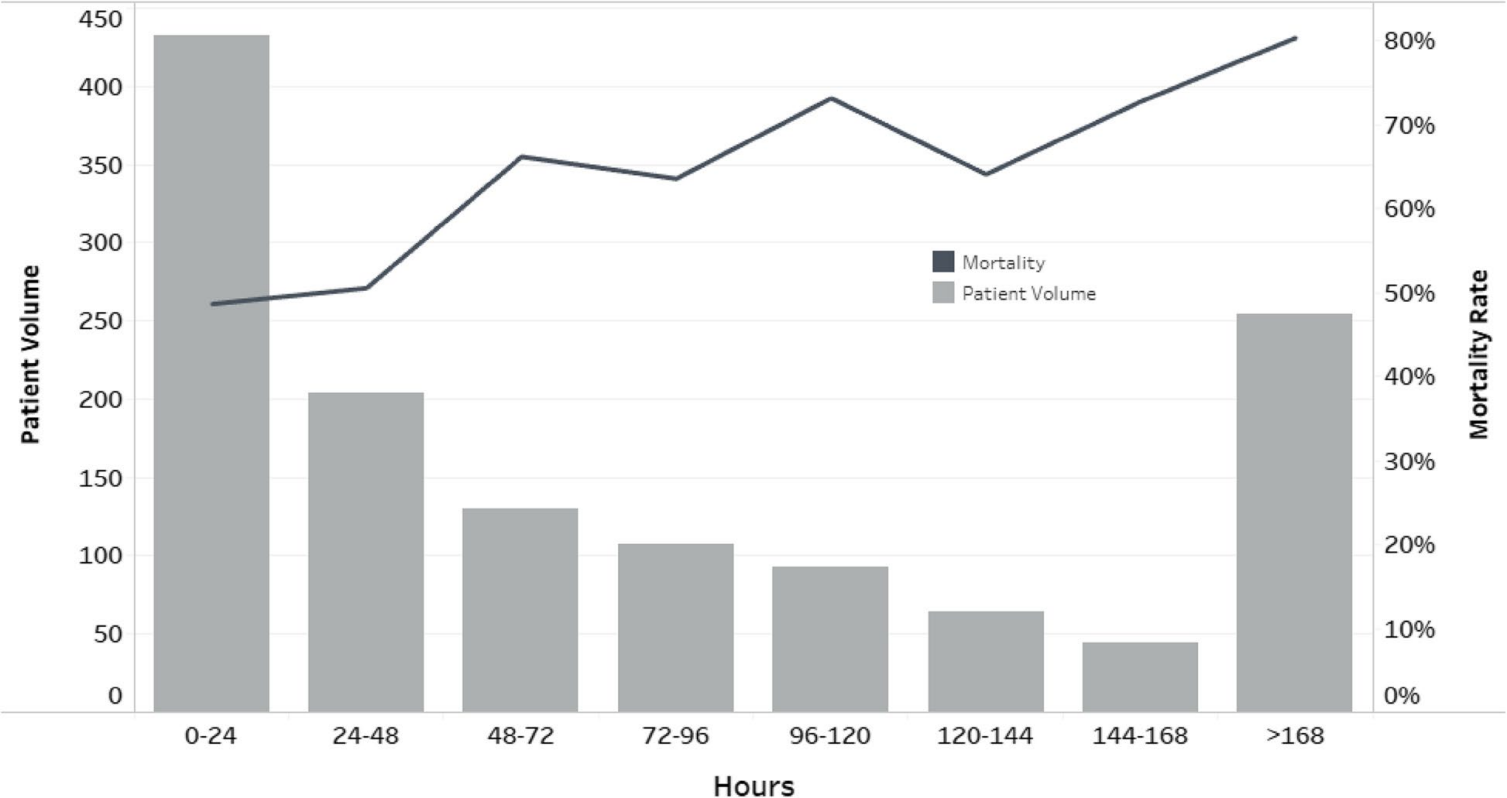
Increased Duration of HFNC Prior to Intubation Associated with High Mortality Rate *Among with acute respiratory failure due to COVID-19 pneumonia who failed HFNC and were intubated, prolonged duration of HFNC prior to intubation was association with increased mortality. Those intubated within 24 h of HFNC had a 50% in-hospital mortality rate. Those who were on HFNC for more than seven days before intubation had a mortality rate of 80%*

A total of 2720 patients with ARF secondary to COVID-19 pneumonia were treated with HFNC. Of these, 1392 (51%) were successfully treated with HFNC alone and 1328 (49%) failed HFNC and were intubated (HFNC to IMV). Among the 1328 patients who were intubated, 311 (23.4%) first transitioned to NiPPV before ultimately being intubated. Of note, all 311 patients transitioned to NiPPV were intubated. Patients treated with HFNC alone had lower mortality rate comparing to patients with HFNC to IMV (17% vs 61%, *p* < 0.0001, OR 7.67, 95%CI [6.42, 9.16], unadjusted). Among those who failed HFNC and were subsequently intubated, the average age was 64 years old. The majority were men (59.5%) and majority (84.6%) had at least one of the comorbidities of COPD, HF, DM, CKD or HTN (Table 1). A Kaplan-Meier survival curve stratified as intubation after 24 h versus intuabtion within 24 h clearly shows a seperation of the curves at the 24-h mark with better survival favoring those intubated within 24 h. Log-Rank test and LR test were statistically significant [SUPPLEMENTAL FIGURE 1].  Those who were intubated within 24 h had the lowest in-hospital mortality (48.6%) and mortality steadily increased with increasing duration of HFNC prior to intubation. Patients on HFNC for greater than 7 days prior to intubation had an in-hospital mortality of 80% [Figure 1]. Based on the above findings, we chose a cutoff of 24 h for duration of HFNC prior to ventilation for comparison. Table 1 compared the covariates: age, sex, comorbidities (COPD, HF, DM, CKD, hypertension), and baseline laboratory values by duration of HFNC (less than 24 h vs. greater than 24 h). Patients who were intubated within 24 h of initiation of HFNC were significantly younger (63 years vs. 65 years old, *p* < 0.001), and had a higher proportion of heart failure diagnosis (26% vs 18%, *p* < 0.01) compared to those intubated after 24 h. The other covariates were similar in both groups.

**Table 1:** Comparison by duration of HFNC (less than 24 h versus greater than 24 h) for patients with ARF due to COVID-19 pneumonia who failed HFNC and were subsequently intubated

	**LESS THAN 24H**	**OVER 24H**	**TOTAL**	***P* VALUES**
TOTAL PATIENT VOLUME	432	896	1328	
AVG. AGE (STD DEV)	63 (14.9)	65 (13)	64 (13.68)	0.0002
SEX (MALE), N (%)	246 (56.9)	544 (60.7)	790 (59.5)	0.19
COPD, N (%)	131 (30.3)	301 (33.6)	432 (32.5)	0.23
HF, N (%)	113 (26.2)	170 (19)	283 (21.3)	0.003
DM, N (%)	133 (30.8)	239 (26.7)	372 (28)	0.12
CKD, N (%)	101 (23.4)	211 (23.6)	312 (23.5)	0.95
HTN, N (%)	300 (69.4)	633 (70.7)	933 (70.3)	0.65
≥ 1 COMORBIDITY, N (%)	372 (86.1)	752 (83.9)	1124 (84.6)	0.30
TROPONIN (> 28NG/L), N (%)	93 (21.5)	161 (18)	254 (19.1)	0.12
CREATININE (> 1.5 MG/DL), N (%)	85 (19.7)	168 (18.8)	253 (19.1)	0.69
CRP (> 100 MG/L), N (%)	132 (30.6)	275 (30.7)	407 (30.7)	0.96
LYMPHOCYTES (< = 1 ΜL), N (%)	243 (56.3)	545 (60.8)	788 (59.3)	0.32
NT-PROBNP (> 88PG/ML), N (%)	132 (30.6)	269 (30)	401 (30.2)	0.84

Table 1. Comparison of covariates: age, sex, comorbidities (COPD, HF, DM, CKD, hypertension), baseline laboratory values (CRP [> 100 mg/L or <  = 100 mg/L], Troponin I [> 28 ng/L or <  = 28 ng/L], NT–proBNP [> 88 pg/mL or <  = 88 pg/mL], Lymphocyte Count [> = 1.0 µL or < 1.0 µL] and Creatinine [< = 1.5 mg/dL or > 1.5 mg/dL]), by duration of HFNC (less than 24 h vs. greater than 24 h)

Based on the results of the univariate analyses, sex, age, DM, hypertension, CKD, troponin, serum creatinine and HFNC duration were identified as potential factors for increased in-hospital mortality [Table 2] and were included in the subsequent multivariable logistic regression model.

**Table 2:** Univariate analysis for 30-day in-hospital mortality among patients with ARF due to COVID-19 pneumonia who failed in HFNC and were subsequently intubated

		**MORTALITY (%)**	**OR (95% CI)**	***P* VALUES**
**SEX**	Male	64.7	1.44 (1.15, 1.80)	0.0013
	Female	56		
**AGE (YEARS)**	≥ 50	66.5	4.42 (3.19, 6.10)	< .0001
	18—49	31		
**COPD**	Yes	60.7	0.97 (0.77, 1.23)	0.8
	No	61.4		
**HF**	Yes	64.3	1.19 (0.90, 1.56)	0.22
	No	60.3		
**DM**	Yes	66.4	1.37 (1.06, 1.76)	0.014
	No	59.1		
**HTN**	Yes	62.6	1.23 (0.96, 1.56)	0.095
	No	57.7		
**CKD**	Yes	71.8	1.85 (1.41, 2.44)	< .0001
	No	57.9		
**TROPONIN (NG/L)**	> 28	70.9	1.7 (1.26, 2.29)	0.0004
	≤ 28	58.9		
**CREATININE (MG/DL)**	> 1.5	75.1	2.2 (1.61, 2.99)	< .0001
	≤ 1.5	57.9		
**CRP (MG/L)**	> 100	59	0.88 (0.69, 1.11)	0.28
	≤ 100	62.1		
**LYMPHOCYTES (ΜL)**	≤ 1	62.2	0.82 (0.63, 1.07)	0.14
	> 1	57.4		
**NT-PROBN (PG/ML)**	> 88	63.6	1.16 (0.91, 1.48)	0.23
	≤ 88	60.1		
**HFNC DURATION**	> 24 h	67.2	2.16 (1.71, 2.74)	< .0001
	≤ 24 h	48.6		

Table 2. Univariate analysis shows that among patients with COVID-19 ARF who failed HFNC and were intubated; male sex, older age, prior diagnosis of diabetes mellitus (DM), a history of chronic kidney disease (CKD), elevated high sensitivity troponin, elevated serum creatinine and prolonged duration of HFNC prior to intubation were all associated with increased in-hospital mortality

When adjusted for the covariates, HFNC duration less than 24 h prior to intubation was significantly associated with reduced mortality [Table 3]**.** When stratified by age greater than or equal to 50 years old or 18–49 years old, HFNC duration less than 24 h prior to intubation remained significantly associated with reduced mortality [SUPPLEMENTAL TABLES 1 and 2]**.**

**Table 3:** Multivariate analysis for 30-day in-hospital mortality among patients with ARF due to COVID-19 pneumonia who failed in HFNC and were subsequently intubated

**COVARIATES**		**OR (95% CI)**	***P*-VALUES**
SEX	Male vs Female	1.34 (1.06, 1.70)	0.016
AGE	≥ 50 vs 18 ~ 49	3.92 (2.79, 5.50)	< .0001
DM	Yes, vs No	0.92 (0.65, 1.26)	0.54
CKD	Yes, vs No	1.50 (1.04, 2.18)	0.03
HTN	Yes, vs No	1.02 (0.78, 1.32)	0.91
TROPONIN (NG/L)	> 28 vs ≤ 28	1.30 (0.92, 1.82)	0.14
CREATININE (MG/DL)	> 1.5 vs ≤ 1.5	1.65 (1.14, 2.38)	0.008
CRP	> 100 vs < = 100	0.83 (0.64, 1.08)	0.16
NT-PROBNP	> 88 vs < = 88	0.91 (0.70, 1.19)	0.51
HFNC DURATION	> 24 h vs ≤ 24 h	2.08 (1.63, 2.67)	< .0001

Table 3, multivariate logistic regression with the covariates sex, age, DM, CKD, HTN, serum troponin, serum creatinine, CRP, NT-PROBNP, and HFNC duration included in the final model based on previously published associations and the univariate logistic regression results

## Discussion

Our study found that among patients with acute respiratory failure due to COVID-19 pneumonia who were initially treated with HFNC but subsequently intubated, delayed intubation beyond twenty-four hours was associated with an increased in-hospital mortality. Initial concerns about potential aerosolization of SARS-CoV-2 viral particles and healthcare personnel infection limited the use of HFNC for patients presenting with acute respiratory failure (ARF) due to COVID-19 pneumonia early in the pandemic [2–6]. However, as infectious risks were better understood, hospital strain escalated, and clinicians gained more experience with HFNC, use of this modality increased. We found that HFNC was successful as a stand-alone respiratory support therapy in 51% of COVID patients receiving this as initial therapy for ARF. Male sex, a history of chronic kidney disease or an elevated baseline serum creatinine (greater than 1.5 mg/dl) were also associated with increased in-hospital mortality (SUPPLEMENTAL TABLE 1). Both factors have been consistently associated with increased mortality from COVID-19 [27, 28].

In the 49% of patients who failed, increased duration of HFNC prior to intubation was associated with worse outcomes. This study adds important evidence to inform optimal use of this therapy and is an important contribution to the growing literature on HFNC. In this study, we sought to answer the question of optimal timing of intubation among those patients who are failing a trial of HFNC. Previous studies have investigated the predictors of HFNC success or failure [29–32]. Generally, old age, a low ROX index at 4 h or at 12 h of HFNC, and high levels of inflammatory markers such as serum ferritin, and CRP predict failure of HFNC [32]. We speculated that among those patients with severe COVID-19 pneumonia and ARF requiring advanced respiratory support, poor compliance of the respiratory system and increased work of breathing, prolonged use of HFNC might exacerbate patient self-inflicted lung injury (SILI) [23]. In this subgroup of patients, early intubation, and lung protective ventilatory strategies might be more beneficial. We confirmed that among those patients with ARF due to COVID-19 pneumonia undergoing a trial of HFNC, intubation and IMV at the twenty-four-hour mark was better than prolonging HFNC. There was a trend of increasing mortality with increasing days on HFNC prior to intubation such that by day 7 of HFNC, failure was associated with 80% mortality. Our results contrast with the findings of Chandel et al. who did not find a statistically significant difference between early intubation after HFNC failure (within 48 h) and later intubation (after 48 h) [33]. This is likely due to low statistical power in their study. Our sample size with 2720 patients was ten times higher. The mortality after late failure of HFNC was 53.2% and only 39.3% among those who failed HFNC early [33]. In our study, those who were intubated within 24 h of failing HFNC had a mortality rate of 48.6% which is much higher than the 39.3% reported by Chandel and colleagues. This probably reflects the severity of disease in our cohort. We also showed a positive correlation with increasing delay of intubation up to one week associated with increased odds of in-hospital mortality [Fig. 1].

In our cohort of 2720 patients, 51% were successfully treated with HFNC alone. This represents a substantial population of ARF patients who would otherwise have required invasive mechanical ventilation. Therefore, during the peak of the pandemic, use of HFNC significantly contributed to relieve hospital systems from capacity strain. In our hospital system, some low-risk patients (younger and with fewer comorbidities) with ARF due to COVID-19 pneumonia were managed with HFNC outside of the intensive care unit (ICU), in consultation with a pulmonary critical care. Our study suggests that this may be a safe approach in carefully selected patients, but that clinicians caring for such patients should consider duration of HFNC as an important risk factor for adverse outcomes. Future prospective studies should assess the feasibility and safety of using HFNC as a substitute for IMV in select patient populations to inform optimal utilization of this therapy.

The strengths of our study include the use of a cohort with detailed phenotyping. We initially included key variables that potentially affect risk of mortality and progression to intubation such as age, sex, comorbidities (chronic obstructive pulmonary disease [COPD], heart failure [HF], Diabetes mellitus [DM], chronic kidney disease [CKD], hypertension[HTN]), baseline laboratory values (C Reactive Protein [CRP] (> 100 mg/L or <  = 100 mg/L), Troponin I (> 28% or <  = 28%), NT–proBNP (> 88 pg/mL or <  = 88 pg/mL), Lymphocyte Count (> = 1.0 µL or < 1.0 µL) and Creatinine (< = 1.5 mg/dL or > 1.5 mg/dL). After multivariate logistic regression adjusting for several of these variables, duration of HFNC beyond 24 h remained significantly associated with increased in-hospital mortality.

The limitations of our study include the retrospective nature of the cohort such that we could not factor all the variables used by the bedside providers when deciding choice of advanced respiratory support and timing of intubation for those failing a trial HFNC. Use of the ROX index to guide decisions about intubation was not uniformly applied and we did not have the serial data on ROX index for majority of the patients.

## Conclusions

Our retrospective analysis shows that patients with severe acute respiratory failure due to COVID-19 pneumonia who fail a trial of HFNC, intubation within the first 24 h of failure is associated with a survival benefit.

## Supplementary Information

**Additional file 1: Supplemental Figure 1.** Kaplan-Meier survival curve stratified as intubation after 24 hours versus intubation within 24 hours shows a separation of the curves at the 24-hour mark with better survival favoring those intubated within 24 hours. Log-Rank test and LR test were statistically significant.

**Additional file 2: SUPPLEMENTAL TABLE 1 **Multivariate analysis for 30-day in-hospital mortality among patients older than 50 years with ARF due to COVID-19 pneumonia who failed in HFNC and were subsequently intubated

**Additional file 3: SUPPLEMENTAL TABLE 2** Multivariate analysis for 30-day in-hospital mortality among patients 18 to 49 years old with ARF due to COVID-19 pneumonia who failed in HFNC and were subsequently intubated.

## Data Availability

The datasets used and/or analyzed during the current study are available from the corresponding author on reasonable request.
