# Supplementary material for: Delayed intubation associated with in-hospital mortality in patients with COVID-19 respiratory failure who fail heated and humified high flow nasal canula
Source: BMC Anesthesiol. 2023 Jul 12;23:234. doi: 10.1186/s12871-023-02198-7 (PMC10337200; doi:10.1186/s12871-023-02198-7)
Supplement: Supplementary file 1 — Additional file 1: Supplemental Figure 1. Kaplan-Meier survival curve stratified as intubation after 24 hours versus intubation within 24 hours shows a separation of the curves at the 24-hour mark with better survival favoring those intubated within 24 hours. Log-Rank test and LR test were statistically significant. [file 12871_2023_2198_MOESM1_ESM.pdf]

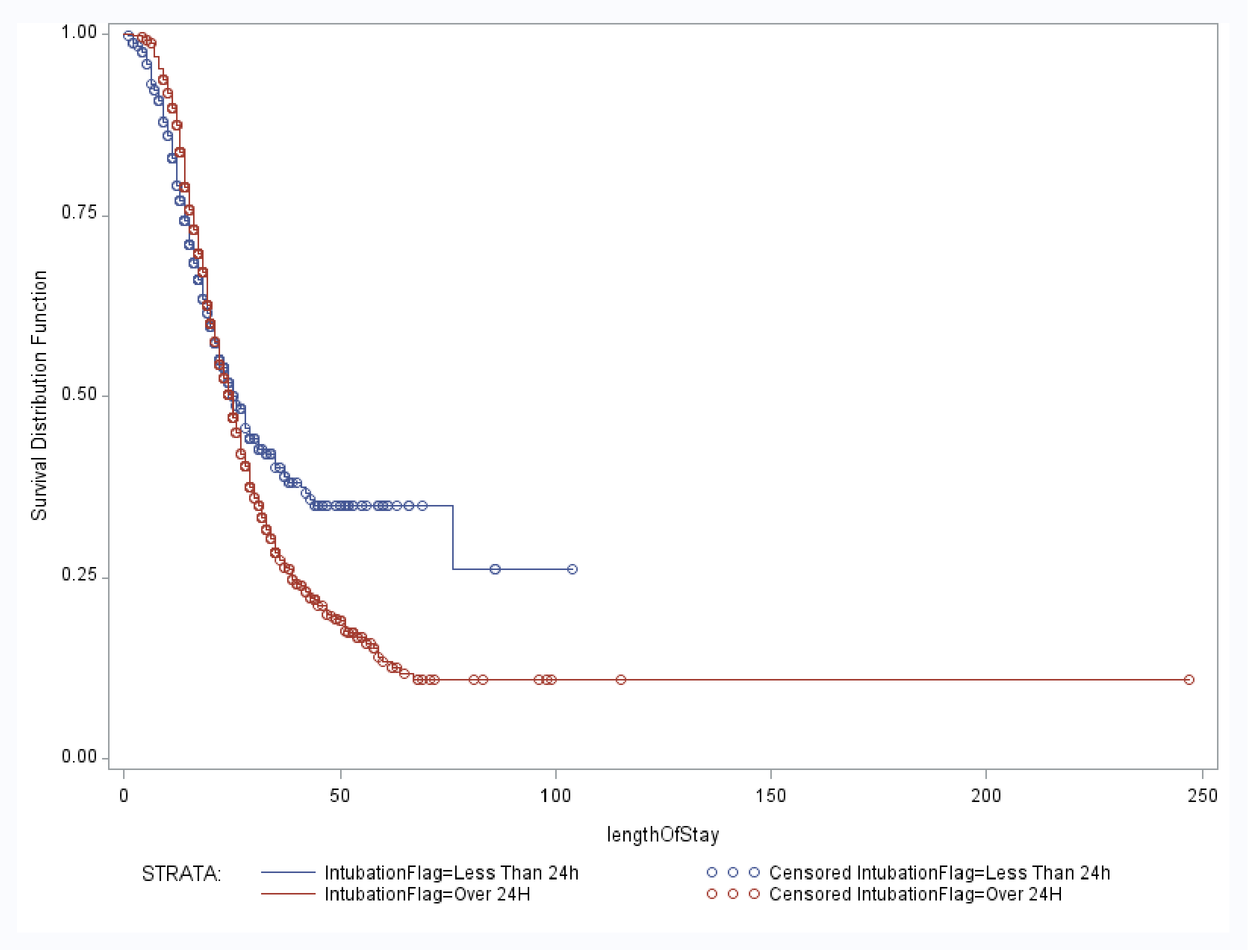

Test of Equality over Strata			
Test	Chi-Square	DF	Pr > Chi-Square
Log-Rank	4.3446	1	0.0371
Wilcoxon	0.4428	1	0.5058
-2Log(LR)	6.5564	1	0.0105

Figure Legend:

Supplemental Figure 1. Kaplan-Meier survival curve stratified as intubation after 24 hours versus intubation within 24 hours shows a separation of the curves at the 24-hour mark with better survival favoring those intubated within 24 hours. Log-Rank test and LR test were statistically significant.
