# Supplementary material for: Delayed intubation associated with in-hospital mortality in patients with COVID-19 respiratory failure who fail heated and humified high flow nasal canula
Source: BMC Anesthesiol. 2023 Jul 12;23:234. doi: 10.1186/s12871-023-02198-7 (PMC10337200; doi:10.1186/s12871-023-02198-7)
Supplement: Supplementary file 2 — Additional file 2: SUPPLEMENTAL TABLE 1 Multivariate analysis for 30-day in-hospital mortality among patients older than 50 years with ARF due to COVID-19 pneumonia who failed in HFNC and were subsequently intubated [file 12871_2023_2198_MOESM2_ESM.docx]

**SUPPLEMENTAL TABLE 1** Multivariate analysis for 30-day in-hospital mortality among patients older than 50 years with ARF due to COVID-19 pneumonia who failed in HFNC and were subsequently intubated

| covariates |  | OR (95% CI) | P-value |
| --- | --- | --- | --- |
| Sex | Male vs Female | 1.33 (1.03, 1.71) | 0.032 |
| DM | Yes, vs No | 0.91 (0.64, 1.30) | 0.60 |
| CKD | Yes, vs No | 1.50 (1.01, 2.24) | 0.05 |
| HTN | Yes, vs No | 1.03 (0.77, 1.38) | 0.84 |
| Troponin (ng/L) | >28 vs ≤ 28 | 1.20 (0.85, 1.71) | 0.30 |
| Creatinine (mg/dL) | >1.5 vs ≤ 1.5 | 1.58 (1.07, 2.33) | 0.021 |
| CRP | >100 vs <=100 | 0.87 (0.66, 1.14) | 0.31 |
| NT-PROBNp | >88 vs <=88 | 0.92 (0.69, 1.22) | 0.55 |
| HFNC Duration | > 24h vs ≤ 24h | 1.93 (1.48, 2.52) | <.0001 |

SUPPLEMENTAL TABLE 1. Multivariate logistic regression for patients older than 50 years old with the covariates sex, age, DM, CKD, HTN, serum troponin, serum creatinine, CRP, NT-PROBNP, and HFNC duration included in the final model based on previously published associations and the univariate logistic regression results.
