# Supplementary material for: Delayed intubation associated with in-hospital mortality in patients with COVID-19 respiratory failure who fail heated and humified high flow nasal canula
Source: BMC Anesthesiol. 2023 Jul 12;23:234. doi: 10.1186/s12871-023-02198-7 (PMC10337200; doi:10.1186/s12871-023-02198-7)
Supplement: Supplementary file 3 — Additional file 3: SUPPLEMENTAL TABLE 2 Multivariate analysis for 30-day in-hospital mortality among patients 18 to 49 years old with ARF due to COVID-19 pneumonia who failed in HFNC and were subsequently intubated. [file 12871_2023_2198_MOESM3_ESM.docx]

**SUPPLEMENTAL TABLE 2** Multivariate analysis for 30-day in-hospital mortality among patients 18 to 49 years old with ARF due to COVID-19 pneumonia who failed in HFNC and were subsequently intubated

| covariates |  | OR (95% CI) | P-value |
| --- | --- | --- | --- |
| SEX | Male vs. Female | 1.37 (0.70, 2.71) | 0.36 |
| DM | Yes, vs No | 0.94 (0.36, 2.44) | 0.89 |
| CKD | Yes, vs No | 1.34 (0.47, 1.91) | 0.59 |
| HTN | Yes, vs No | 0.96 (0.48, 1.91) | 0.90 |
| Troponin (ng/L) | >28 vs ≤ 28 | 3.31 (0.93, 11.82) | 0.07 |
| Creatinine (mg/dL) | >1.5 vs ≤ 1.5 | 2.26 (0.70, 7.26) | 0.17 |
| CRP | >100 vs <=100 | 0.56 (0.26, 1.20) | 0.14 |
| NT-PROBNp | >88 vs <=88 | 0.90 (0.37, 2.16) | 0.81 |
| HFNC Duration | > 24h vs ≤ 24h | 3.37 (1.68, 6.76) | 0.0009 |

SUPPLEMENTAL TABLE 2. Multivariate logistic regression for patients 18-49 years old with the covariates sex, age, DM, CKD, HTN, serum troponin, serum creatinine, CRP, NT-PROBNP, and HFNC duration included in the final model based on previously published associations and the univariate logistic regression results.
